# Supplementary material for: Inflammatory factors and risk of lung adenocarcinoma: a Mendelian randomization study mediated by blood metabolites
Source: Front Endocrinol (Lausanne). 2024 Aug 27;15:1446863. doi: 10.3389/fendo.2024.1446863 (PMC11384989; doi:10.3389/fendo.2024.1446863)
Supplement: Supplementary file 1 [file DataSheet1.zip › supplementary files/S2/GCST90200603/forest.pdf]

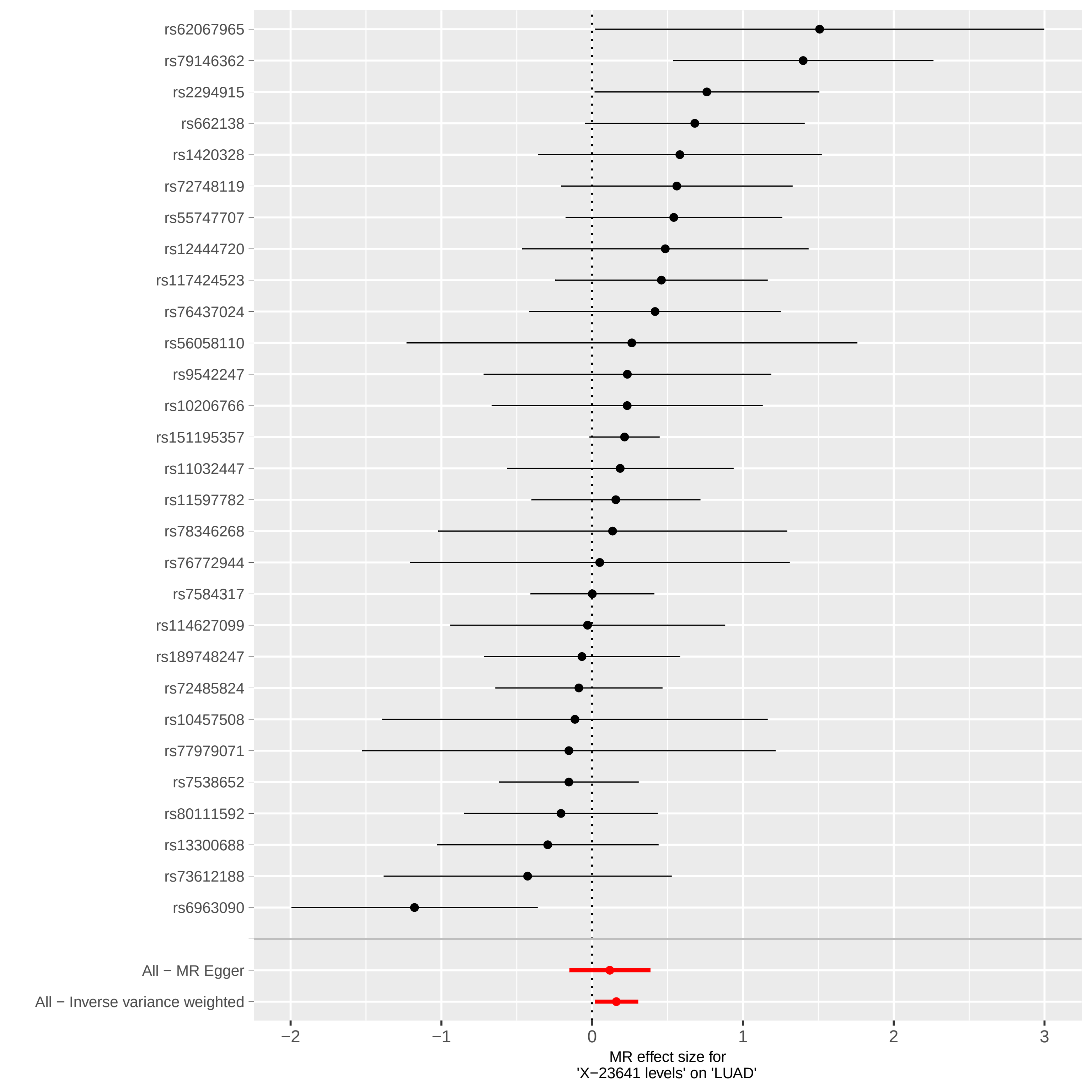

rs62067965
rs79146362
rs2294915
rs662138
rs1420328
rs72748119
rs55747707
rs12444720
rs117424523
rs76437024
rs56058110
rs9542247
rs10206766
rs151195357
rs11032447
rs11597782
rs78346268
rs76772944
rs7584317
rs114627099
rs189748247
rs72485824
rs10457508
rs77979071
rs7538652
rs80111592
rs13300688
rs73612188
rs6963090
All − MR Egger
All − Inverse variance weighted
−2
−1
0
1
2
3
MR effect size for
'X−23641 levels' on 'LUAD'
